# The scenic route: dilated left superior intercostal vein following acute left brachiocephalic venous obstruction

**DOI:** 10.1093/europace/euac120

**Published:** 2023-02-08

**Authors:** Nicholas Y Tan, Abhishek J Deshmukh

**Affiliations:** Department of Cardiovascular Medicine, Mayo Clinic, 200 1st Street Southwest, Rochester, MN 55905, USA; Department of Cardiovascular Medicine, Mayo Clinic, 200 1st Street Southwest, Rochester, MN 55905, USA

A 62-year-old man was scheduled for left-sided permanent pacemaker implantation for post-operative complete heart block. A peripheral venogram showed patency of the axillary and proximal subclavian veins. After access was obtained, the micropuncture wire did not follow the typical course expected of a left brachiocephalic vein. A venogram was therefore performed through a 5 Fr short sheath—this revealed a U-shaped course with eventual drainage into the superior vena cava (*Panels A* and *B*).

These represent a dilated left superior intercostal vein that drains into the accessory hemiazygous vein, followed by the azygous vein and finally the superior vena cava. The prominence of this vessel can occur with congenital or acquired obstruction of the proximal veins. In this case, a dialysis catheter-associated thrombus likely caused acute left brachiocephalic vein occlusion and consequent increase in collateral flow.

**Figure:**
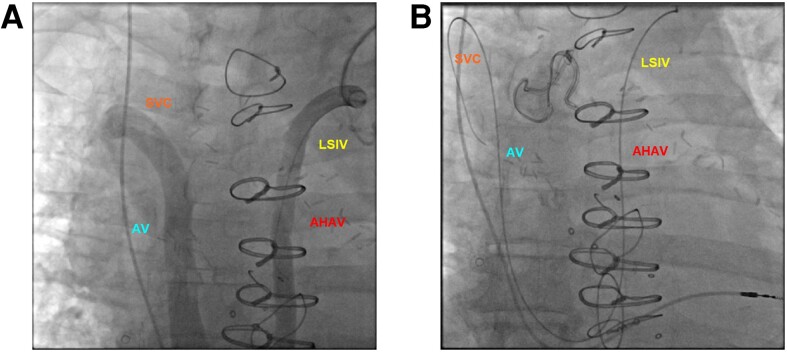


Pursuing left-sided device implantation was not deemed feasible, given the long and meandering venous course visualized. Hence, a dual-chamber pacemaker was implanted on the right side instead. The invasive electrophysiologist should be aware of these uncommon but impressive venous anomalies in the planning and execution of cardiac device implantations.

The full-length version of this report can be viewed at: https://www.escardio.org/Education/E-Learning/Clinical-cases/Electrophysiology.

